# Long Non-coding RNA Maternally Expressed 3 Increases the Expression of Neuron-Specific Genes by Targeting miR-128-3p in All-Trans Retinoic Acid-Induced Neurogenic Differentiation From Amniotic Epithelial Cells

**DOI:** 10.3389/fcell.2019.00342

**Published:** 2019-12-23

**Authors:** Yuhua Gao, Ranxi Zhang, Guanghe Wei, Shanshan Dai, Xue Zhang, Wancai Yang, Xiangchen Li, Chunyu Bai

**Affiliations:** ^1^Institute of Precision Medicine, School of Clinical Medicine, Jining Medical University, Jining, China; ^2^Institute of Animal Sciences, Chinese Academy of Agricultural Sciences, Beijing, China; ^3^Department of Spine Surgery, Qingdao Municipal Hospital, Qingdao, China; ^4^Department of Pathology, University of Illinois at Chicago, Chicago, IL, United States; ^5^College of Animal Science and Technology, College of Veterinary Medicine, Zhejiang A&F University, Lin’an, China

**Keywords:** AECs, neurogenic differentiation, ATRA, miRNA, lncRNA

## Abstract

MicroRNA (miR)-128-3p is a brain-enriched miRNA that participates in the regulation of neural cell differentiation and the protection of neurons, but the mechanisms by which miR-128-3p regulates its target and downstream genes to influence cell fate from adult stem cells are poorly understood. In this study, we show down-regulation of miR-128-3p during all-trans retinoic acid (ATRA)-induced neurogenic differentiation from amniotic epithelial cells (AECs). We investigated miR-128-3p in both the Notch pathway and in the expression of neuron-specific genes predicted to be involved in miR-128-3p signaling to elucidate its role in the genetic regulation of downstream neurogenic differentiation. Our results demonstrate that miR-128-3p is a negative regulator for the transcription of the neuron-specific genes β III-tubulin, neuron-specific enolase (NSE), and polysialic acid-neural cell adhesion molecule (PSA-NCAM) via targeting Jagged 1 to inhibit activation of the Notch signaling pathway. We also used bioinformatics algorithms to screen for miR-128-3p interactions with long non-coding (lnc) RNA and circular RNA as competing endogenous RNAs to further elucidate underlying down-regulated molecular mechanisms. The lncRNA maternally expressed 3 is up-regulated by the ATRA/cAMP/CREB pathway, and it, in turn, is directly down-regulated by miR-128-3p to increase the amount of neuron differentiation. Endogenous miRNAs are, therefore, involved in neurogenic differentiation from AECs and should be considered during the development of effective cell transplant therapies for the treatment of neurodegenerative disease.

## Introduction

Amniotic epithelial cells (AECs) are derived from the epiblast of the amniotic membrane and exhibit biological characteristics similar to embryonic stem cells, which have the capacity for differentiation into all three germ layers, as well as neural cells. Therefore, AECs have potential use in the recovery of injured nervous tissues. All-trans retinoic acid (ATRA) is the active ingredient of Vitamin A and plays an important role in the development of the nervous system by providing trophic effects that support the proliferation, differentiation, and maintenance of neural cells. ATRA affects the differentiation of neural cells derived from stem cells, including embryonic stem cells, mesenchymal stem cells, induced pluripotent stem cells, and AECs (Mark et al., 2015; Samarut et al., 2015; Davenport et al., 2016).

MicroRNAs (miRNAs) are a major group of endogenous, non-coding, small ribonucleotides (18–25 nt long) present in invertebrates, vertebrates, and plants that play important roles in the regulation of gene expression through the 3′-untranslated region (UTR) binding of specific messenger RNAs (mRNAs) to interfere with transcription (Bofill-De Ros et al., 2019). The role of miRNAs in the differentiation and development of the nervous system has received increased attention and importance in recent research, and specific miRNA spatiotemporal expression patterns may be essential for neuron neurogenesis (Volvert et al., 2012; Zhang et al., 2014; Bai et al., 2019). miR-128 is a brain-enriched miRNA that was reported to participate in the regulation of neural cell differentiation. Zhang et al. (2016) demonstrated that miR-128 prevents cortical neural progenitor cells (NPCs) from dividing and supports the specialized development of cells in a mouse model. Removing miR-128 from mouse NPCs enhanced cell division, resulting in less neuron formation (Zhang et al., 2016).

The human miR-128-1 stem-loop sequence contains two mature sequences, miR-128-1-5p and miR-128-3p. miR-128-3p was observed to be neural-protective after Fingolimod (FTY720) (and derivatives) treatment in dopaminergic MN9D cells (Vargas-Medrano et al., 2019). FTY720 is a Food and Drug Administration-approved drug for Parkinson’s disease therapeutics. In the present study, we observed significant down-regulation of miR-128-3p in ATRA-induced neural cell differentiation from AECs. Dramatic positive changes were also observed in the expression of β III-tubulin, a specific neural marker. We investigated downstream miR-128-3p-regulated pathways and demonstrated the effects of those pathways on activation of the Notch signaling pathway related to the influence of neuronal marker expression to better illuminate the roles of miR-128-3p in the transcriptional regulation of neural cell differentiation. Additionally, we analyzed the causes of decreased miR-128-3p expression. We identified the long non-coding RNA (lncRNA) maternally expressed 3 (MEG3) as interacting with miR-128-3p, and previous reports of its up-regulation through a series of gene transcriptions after cAMP pathway activation (Zhao et al., 2006; Zhu et al., 2019). This reinforces our current miR-128-3p biological function proposal.

## Materials and Methods

### Cell Culture and Neural Cell Differentiation

Amniotic epithelial cells were obtained from human amniotic membranes using enzyme digestion (Gao et al., 2016) and were cultured in Dulbecco’s modified Eagle’s medium/Ham’s F-12 supplemented with 10% fetal bovine serum (Gibco, Carlsbad, CA, United States), 10 ng/ml epidermal growth factor, 10 ng/ml basic fibroblast growth factor, and 55 μM β-Mercaptoethanol. AECs were plated in six-well plates for neural differentiation and then 5 × 10^–5^ M ATRA was added to AEC cultures. After culturing for 18 days, the cells were harvested to analyze the expression of specific markers.

### Reverse Transcription Quantitative PCR

miR-128-3p transcription levels were analyzed using reverse transcription quantitative (RT-q)PCR after neural differentiation. miRNAs were isolated from induced AECs with the miRcute miRNA Isolation Kit (Tiangen Biotech, Beijing, China), and pure miRNAs were poly(A)-tailed and reverse-transcribed with the miRcute miRNA First-strand cDNA Synthesis Kit (Tiangen Biotech, Beijing, China). qPCR was performed with the SYBR Green-based miRcute miRNA qPCR Detection Kit (Tiangen Biotech, Beijing, China) on an ABI 7500 Real-Time PCR system (Thermo Fisher Scientific, Waltham, MA, United States) according to the manufacturer’s instructions. miR-128-3p and U6 primers were obtained from Tiangen Biotech, Beijing, China.

The expression of genes upstream and downstream of miR-128-3p [β III-tubulin, glial fibrillary acidic protein (*GFAP*), Jagged 1 (*JAG1*), and *MEG3*] was also tested using RT-qPCR. Total RNA was isolated from AECs and induced AECs using TRIzol reagent (Gibco, Carlsbad, CA, United States). It was then reverse-transcribed and used for qPCR analysis with the RNA PCR kit version 3.0 (Takara, Dalian, China) on an Applied Biosystems 7500 Real-Time PCR system. Reactions were performed in 20-μl volumes containing 7 μl of dd H_2_O, 10 μl of SYBR premix Ex Taq buffer, 0.8 μM each of forward and reverse primers, 0.4 μl of ROX Reference Dye, and 1 μl of template cDNA. PCR conditions were 30 PCR cycles. Each experiment was performed in duplicate in 96-well plates and repeated three times. The mRNA gene expression cycle threshold value from each sample was calculated and normalized to internal controls using glyceraldehyde-3-phosphate dehydrogenase (GAPDH), and relative values were plotted. Primers are listed in Supplementary Table S1.

### Immunofluorescence

Amniotic epithelial cells and induced AECs were fixed using 4% paraformaldehyde for 15 min and then permeabilized with 0.01% Triton X-100 for 30 min. After rinsing three times with phosphate-buffered saline (PBS), cells were blocked with 4% bovine serum albumin for 1 h and then incubated with primary antibodies [GFAP, 1:500; β III-tubulin, 1:200; Notch intracellular domain (NICD), 1:500; HES5, 1:500; CREB, 1:200] overnight in a humidified chamber at 4°C. All antibodies were purchased from Abcam (Cambridge, MA, United States). Cells were then incubated with Cy5 or FITC-labeled secondary antibodies at room temperature for 1 h the following day. Immunofluorescent data were obtained using a confocal optical system (Nikon TE2000, Tokyo, Japan).

### Flow Cytometry

To determine the level of β III-tubulin and GFAP expression after neural differentiation, cells were analyzed using an FC500 flow cytometer (Beckman Coulter, Atlanta, GA, United States). Briefly, cells were collected, blocked, and labeled with FITC- or Cy5-conjugated antibodies against β III-tubulin or GFAP as per manufacturer instructions. Data were analyzed with CXP software (Beckman Coulter, Atlanta, GA, United States). Mean fluorescence intensity was determined after the subtraction of a negative control (normal AECs).

### Western Blotting

The expression of proteins encoded by genes up- and downstream of miR-128-3p was detected by Western blot analysis. Cells were lysed with the NP40 Extraction Reagent (CWBio, Beijing, China) and supplemented with a protease inhibitor (CWBio, Beijing, China). The bicinchoninic acid assay was used to measure protein concentrations. Extracted proteins were mixed with loading buffer and subjected to sodium dodecyl sulfate–polyacrylamide gel electrophoresis, followed by transfer to 0.2-μm nitrocellulose membranes. Primary antibodies (GFAP, 1:500; β III-tubulin, 1:400; NICD, 1:200; HES5, 1:500; CREB, 1:500, GAPDH, 1:5000; and histone, 1:5000) and horseradish peroxidase (HRP)-labeled secondary antibodies (1:10,000) were purchased from Abcam, Cambridge, MA, United States. Proteins were visualized with the Pierce ECL Western blotting substrate (CWBio, Beijing, China) for HRP. GAPDH and histone were used as internal controls.

### Site-Directed Mutagenesis

The PCR-based Fast Site-Directed Mutagenesis Kit (Tiangen Biotech, Beijing, China) was used to mutate the base sequence. Primers containing the appropriate base substitutions are listed in Supplementary Table S1. PCR products were digested with the restriction enzyme *Dpn*I (Thermo Fisher Scientific, Waltham, MA, United States) at 37°C for 8 h and then transformed into competent *E. coli* cells. Mutation sites were confirmed using Sanger sequencing.

### Luciferase Reporter Assays

We constructed a pGL3.0-Luc plasmid with target 3′-UTRs using firefly luciferase reporter vectors to test whether predicted miR-128-3p-binding sites in the 3′-UTR of the target were responsible for silencing expression. A mutation at nucleotide position 4 of the miRNA seed sequence in each 3′-UTR was generated with the Fast Site-Directed Mutagenesis Kit according to the manufacturer’s instructions. Constructs containing mutated target (MUT) 3′-UTRs were used as the test group. Lipofectamine 3000 (Gibco, Carlsbad, CA, United States) was used to transfect HEK293T cells with a mixture of firefly luciferase reporter plasmids (wild-type plasmid, WT and mutation plasmid, MUT), the miRNA precursor or control, and the *Renilla reniformis* luciferase-encoding plasmid.

### Hes 5-Binding Site Prediction

Putative Hes 5-binding sites within the promoter sequences of neuron-specific enolase (*NSE*), β III-tubulin, and polysialic acid-neural cell adhesion molecule (*PSA-NCAM*) were predicted using JASPAR CORE databases^1^ (Khan et al., 2018), which construct specific binding site weight matrices for binding site prediction. The promoter sequences of *NSE*, β III-tubulin, and *PSA-NCAM* were obtained from the UCSC Genome Browser Gateway and were defined based on the location of the GC box and TATA box.

### Chromatin Immunoprecipitation Assay-PCR

Chromatin immunoprecipitation (ChIP) was performed using the ChIP assay kit (Beyotime Institute of Biotechnology, Beijing, China) to test whether the transcription factor HES5 binds to the promotor regions of neural genes *NSE*, β III-tubulin, and *PSA-NCAM*. ChIP DNA was extracted with a DNA Purification Kit (Beyotime Institute of Biotechnology, Beijing, China), and the purified sample was subjected to qPCR amplification with primers spanning the protein-binding sites (Supplementary Table S1).

### Co-immunoprecipitation

To test for interactions between MEG3 and miR-128-3p, an Argonaute 2 (AGO2)-containing plasmid and the miRNA precursor were co-transfected into AECs. After 3 days, cells were lysed in 1 ml of lysis buffer (CWBio, Beijing, China), lysates were centrifuged, and 1 μg of AGO2 antibodies was added to 500 μl of the supernatant. IP was carried out with a Pierce Co-immunoprecipitation (Co-IP) Kit (Thermo Fisher Scientific, Waltham, MA, United States) according to the manufacturer’s instructions and then MEG3 expression was analyzed in the IP products using qPCR. The empty plasmid (EV) and miRNA-Let 7a served as experimental controls.

### Statistical Analysis

The Student’s *t*-test was used to compare data between the two experimental groups. Statistical significance was defined as ^∗^*P* < 0.05 and ^∗∗^*P* < 0.01. All studies were performed in three separate experiments, each performed in triplicate. All data are expressed as the mean ± standard deviation (SD).

## Results

### miR-128-3p Signature in Neurogenic Differentiation From AECs

**FIGURE 1:**
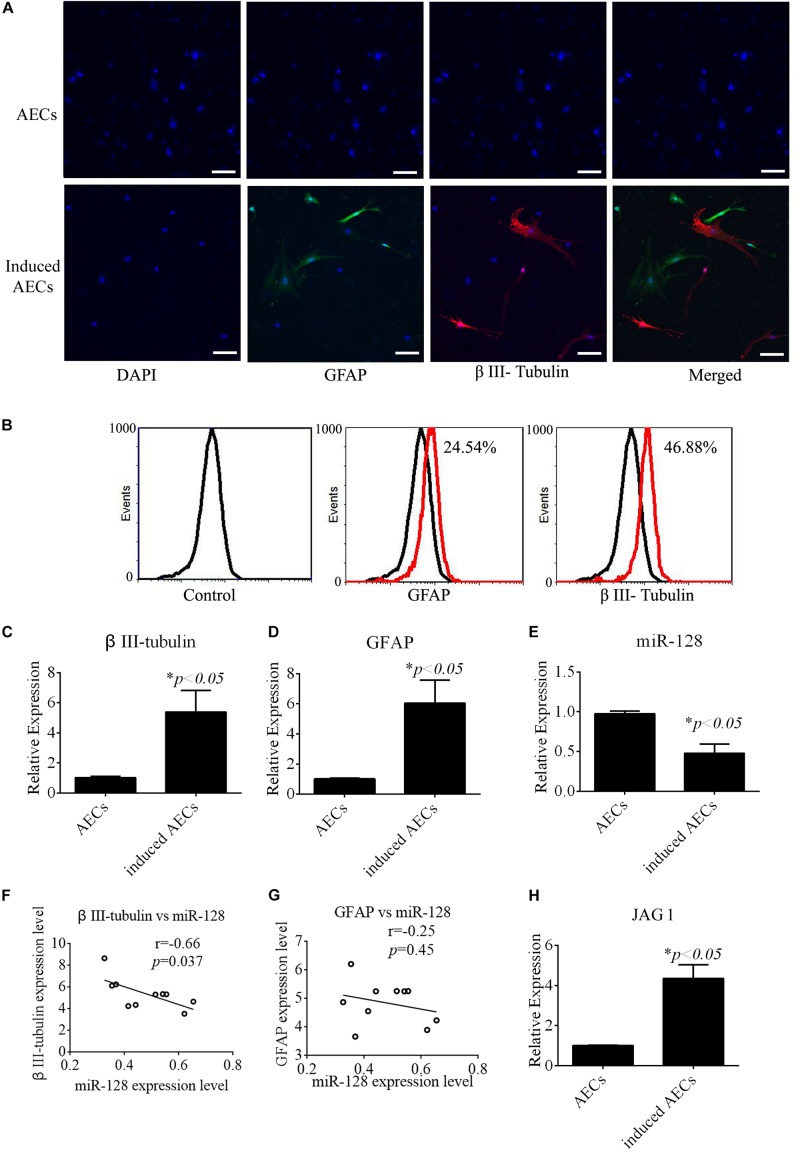
Neurogenic differentiation derived from amniotic epithelial cells (AECs). AECs were induced with all-trans retinoic acid (ATRA) for 18 days. **(A,B)** Neurogenic markers were detected by immunofluorescence staining and flow cytometry. Neuron-specific genes (β III-tubulin) and astrocyte-specific genes (glial fibrillary acidic protein: GFAP) were detected. Scale bar = 100 μm. **(C–E)** RT-qPCR was used to determine the relative expression of β III-tubulin, GFAP, and miR-128-3p in non-induced AECs and induced AECs. **(F,G)** Comparative analysis of miR-128-3p and β III-tubulin or GFAP in neurogenic differentiation. **(H)** The expression of Jagged 1 (JAG1), a putative target for miR-128-3p in neurogenic differentiation, was significantly altered in induced AECs. ^∗^*P* < 0.05.

All-trans retinoic acid affects the differentiation of neural cells from stem cells. The effects of ATRA in neurogenic differentiation from AECs were assessed in this study. AECs induced toward neurogenic differentiation take on a different, elongated neuronal morphology, with apparent large, flat, and multipolar cells. Neurogenic differentiation was evaluated according to the expression level of neuron-specific genes. After treatment with ATRA, expression of the neuron-specific gene β III-tubulin and astrocyte-specific gene *GFAP* was detected using immunofluorescence, flow cytometry, and RT-qPCR. Increased β III-tubulin and GFAP expression levels were detected in ATRA-induced AECs (Figures 1A–D) compared with untreated cells.

miR-128 is a brain-enriched miRNA, and miR-128-3p has been shown to be neuroprotective (Zhang et al., 2016). Therefore, we tested the relative expression levels of miR-128-3p, as well as that of JAG1 protein as a putative target of miR-128-3p predicted using TargetScan Human v7.2^2^, in normal AECs and induced AECs using RT-qPCR (Figures 1E,H). miR-128-3p expression decreased dramatically following neurogenic differentiation, while JAG1 expression was significantly up-regulated. Expression level analysis of miR-128-3p with β III-tubulin showed a significantly negative correlation between normal AECs and induced AECs (*P* < 0.05, Figures 1F,G). These results demonstrate that miR-128-3p plays a critical role in the expression of neuron-specific genes.

### Notch Pathway Activation and Roles in the Expression of Neuron-Specific Genes

**FIGURE 2:**
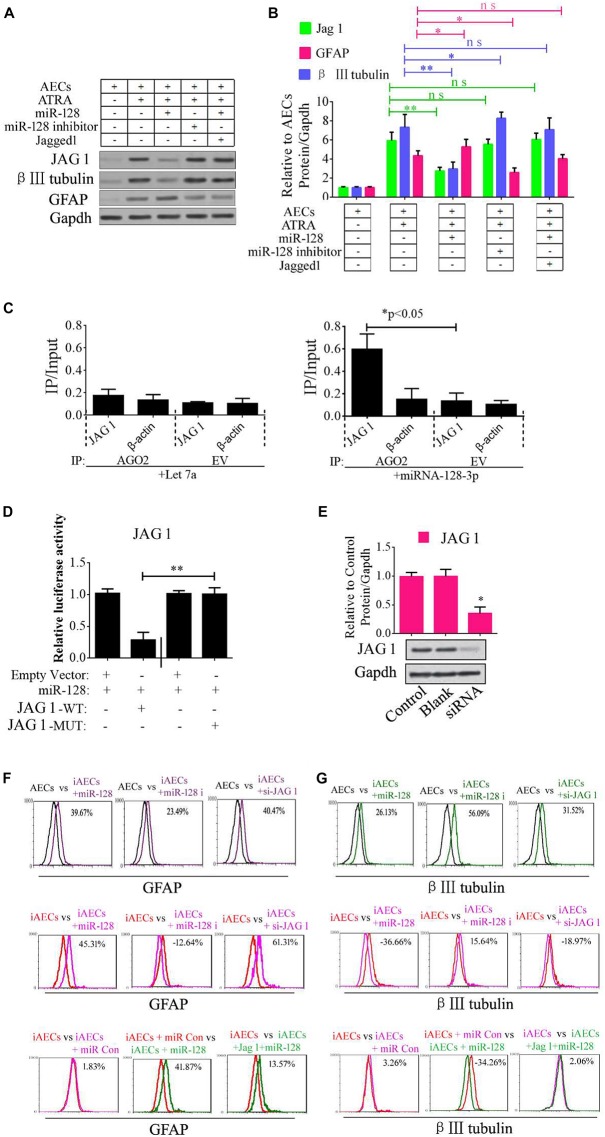
Role of miR-128-3p in neurogenic differentiation from AECs. **(A,B)** Neurocyte markers were tested using Western blotting after AECs were exposed to miR-128-3p, miR-128-3p inhibitor, and Jagged 1, respectively. Protein abundance was analyzed using ImageJ tools. The data revealed that Jagged1 could rescue the β III-tubulin expression after overexpressed miR-128 in AECs. ^∗^*P* < 0.05; ^∗∗^*P* < 0.01; ns, non-significant. **(C)** Immunoprecipitation of Myc-tagged Argonaute 2 (AGO2) from AECs co-transfected with Myc-AGO2 and either miR-128-3p or Let-7a (negative control). The empty vector (EV) served as the Myc-AGO2-related negative control. Jag 1 and β-actin mRNA levels were quantified using qPCR, and the relative immunoprecipitate (IP)/input (cell total RNA) values were plotted. ^∗^*P* < 0.05. **(D)** The effect of miR-128-3p on JAG1 expression was evaluated using luciferase reporter assays. ^∗∗^*P* < 0.01. **(E)** Western blot analysis of JAG1 expression was performed following siRNA targeting. Protein abundance was analyzed using ImageJ tools. Glyceraldehyde-3-phosphate dehydrogenase (GAPDH) was used as an endogenous control and Scramble was used as a control. Western blot images are representative of at least three independent replicates. ^∗^*P* < 0.05. **(F,G)** Neurocyte markers were tested using flow cytometry after induced AECs were exposed to various small RNAs or Jagged 1. iAECs, induced AECs; miR-128 i, miR-128 inhibitor; miR Con, miR-128 mimics control.

Jagged 1 is the canonical ligand for the receptor of notch 1, which interacts with cell surface transmembrane-spanning receptors of the Notch pathway to release a NICD from the membrane tether. The NICD translocates into the nucleus and associates with the CBF1/RBP-Jk/Suppressor of Hairless/LAG-1 (CSL) family of DNA-binding proteins to form a transcriptional activator. This activates the tissue-specific basic helix–loop–helix hairy and enhancer of split (HES) gene family members HES1 and HES5, which enhances transcription of a set of target genes. In our research, we first tested the regulation of miR-128-3p, JAG1, β III-tubulin, and GFAP. miR-128-3p mimics and inhibitor were added to the induced AECs. The protein levels of the abovementioned genes were tested by Western blot analysis, and JAG1 and β III-tubulin expression was shown to dramatically decrease, while GFAP levels were significantly up-regulated after the overexpression of miR-128-3p in induced AECs; the opposite was observed with the addition of an inhibitor, the effect of overexpressed miR-128 in induced AECs was rescued after JAG1 addition (Figures 2A,B).

To determine whether the predicted site in the 3′-UTR of JAG1 is responsible for the silencing of gene expression by miR-128-3p, the AGO2-IP qPCR was used for further analyses. As an essential component of the RNA-induced silencing complex (RISC), the AGO2 protein plays a central role in RNA silencing processes. To determine whether AGO2 serves as a binding platform for JAG1 and miR-128-3p, we performed Myc-AGO2 IP in HEK293T cells containing either an AGO expression vector or an empty vector and transiently co-expressing miR-128-3p or Let-7a (negative control). The binding site of Let-7a was not found in the JAG1 sequence after analysis using bioinformatics algorithms, so Let-7a was used as a negative control in this study. JAG1 levels were analyzed by qPCR of the IP products, and shown to be specifically enriched by more than five- to sevenfold in the presence of AGO2 in miR-128-3p-transfected cells compared with the control (Let-7a-transfected cells, Figure 2C). These data suggest that miR-128-3p interacts with JAG1 in the RISC complex. We next cloned WT and MUT versions of the *JAG1* 3′-UTR region downstream of a luciferase reporter gene and then co-transfected one or the other of these vectors into HEK293T cells with either pre-miR-128 or a control. In cells transfected with pre-miR-128 and pRL-JAG1-WT, luciferase activity significantly decreased relative to that in cells co-transfected with the control precursor or mutated versions of the target (Figure 2D). These results demonstrate that miR-128 directly suppresses the expression of JAG1 through targeting seed sequences in the 3′-UTR of *JAG1*, in agreement with a previous report (Yi et al., 2018). To test the role of JAG1 in neurogenic differentiation, a small interfering RNA (siRNA) of JAG1 was designed, synthesized, and verified (Figure 2E). Then, miR-128-3p mimics, inhibitor, and siRNA-JAG1 were separately added to the induced AECs, and flow cytometry was used to analyze the relative expression of β III-tubulin and GFAP. miR-128-3p and siRNA-JAG1 efficiently prevented β III-tubulin expression, the miR-128-3p inhibitor increased expression, while GFAP had the opposite effect of β III-tubulin (Figures 2F,G).

**FIGURE 3:**
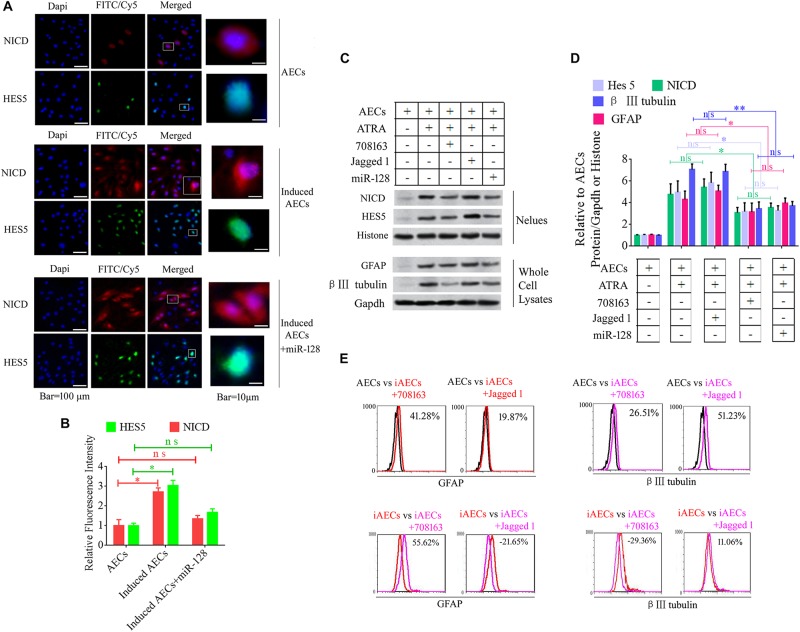
Notch pathway inhibition by miR-128-3p decreases the expression of neurocyte markers in neurogenic differentiation. **(A)** Immunofluorescence staining illustrates activation of the Notch pathway based on the nuclear expression of intercellular activated notch 1 (Notch intracellular domain: NICD) and Hes 5 after ATRA induction and miR-128 addition. **(B)** Fluorescence intensity was analyzed using ImageJ tools. **(C,D)** Western blot data of NICD and HES5 expression after JAG1, BMS-708163, or miR-128 overexpression treatment. Protein abundance was analyzed using ImageJ tools. ^∗^*P* < 0.05; ^∗∗^*P* < 0.01; ns, non-significant. **(E)** Neurocyte markers were tested using flow cytometry in induced AECs exposed to 708163 (an inhibitor of the Notch pathway) or Jagged 1 (activator for the Notch pathway). iAECs, induced AECs.

Notch intracellular domain and HES5 were analyzed using immunofluorescence to test for activation of the Notch pathway after ATRA treatment in AECs. NICD and HES5 levels significantly increased in induced AECs, but the opposite data were observed after overexpressed miR-128 in induced AECs (Figures 3A,B). Avagacestat (BMS-708163) is a potent inhibitor of γ-secretase and NICD and exhibits potency for Notch processing inhibition, so the effect of BMS-708163 and JAG1 was determined by separately adding them to induced AECs. Flow cytometry showed that JAG1 increased the amount of β III-tubulin, while BMS-708163 reduced its expression (Figure 3C). To determine the influence of BMS-708163 or JAG1 on NICD and HES5 in the nucleus, we extracted nuclear protein to test expression. NICD and HES5 expression dramatically increased by JAG1 treatment, while the opposite occurred with BMS-708163 treatment. β III-Tubulin expression increases following activation of the Notch pathway (Figures 3D,E), suggesting that this plays an important role in the expression of neuron-specific genes in AEC neurogenic differentiation derived from AECs, but the mechanisms by which HES5 regulates the expression of neuron-specific genes to influence cell fate remains poorly understood.

**FIGURE 4:**
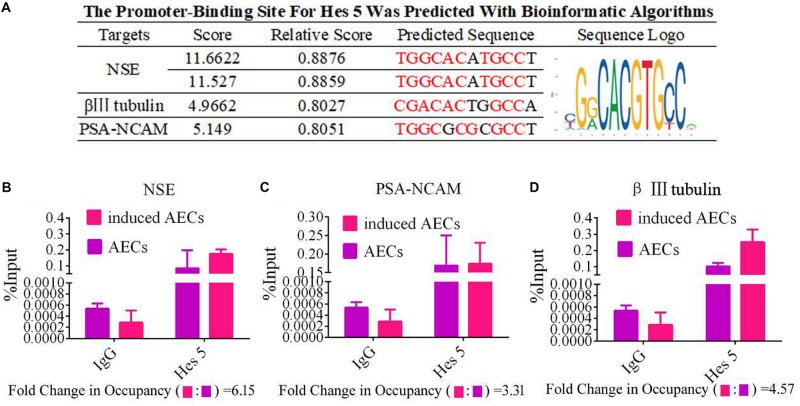
HES5 promotes transcription of the neurocyte marker genes β III-tubulin, *NSE*, and *PSA-NCAM* via physical interactions with their promoters. **(A)** HES5 promoter-binding sites were predicted using bioinformatics algorithms in the promoter region of β III-tubulin, *NSE*, and *PSA-NCAM*. Predicted sequences were calculated according to the relative frequency of promotor sequences, which have the potential to bind Hes 5 but do not completely conform to the sequence logo of Hes 5. Consensus sequences are shown in red. **(B–D)** The amount of HES5 enriched within neurocyte marker gene promoter regions in normal and induced AECs was quantified by ChIP-coupled real-time PCR. The percentage of input was calculated according to threshold cycle values. The negative control for Hes 5 binding sites is shown in the Supplementary Material. Data are presented as the mean ± SD (three independent experiments).

To determine the mechanisms underlying the elevated expression of neuron-specific genes after activation of the Notch pathway, the promoter regions (3 kb upstream) of β III-tubulin, *NSE*, and *PSA-NCAM* were screened using JASPAR tools and multiple HES5-binding sites were found (Figure 4A). To test for physical interactions and to accurately compare the amounts of HES5 enriched in these promoter regions, ChIP coupled with qPCR analysis was performed. Non-specific IgG was used as a negative control, and the input sample (whole lysate prior to ChIP being performed) was used as a positive control. As shown in Figures 4B–D, the fold change in occupancy was higher in induced AECs than in normal AECs, with observed fold changes of 4.57, 6.15, and 3.31 for β III-tubulin, *NSE*, and *PSA-NCAM*, respectively.

### LncRNA MEG3 as a Competing Endogenous RNA to Influence the Expression of miR-128-3p

**FIGURE 5:**
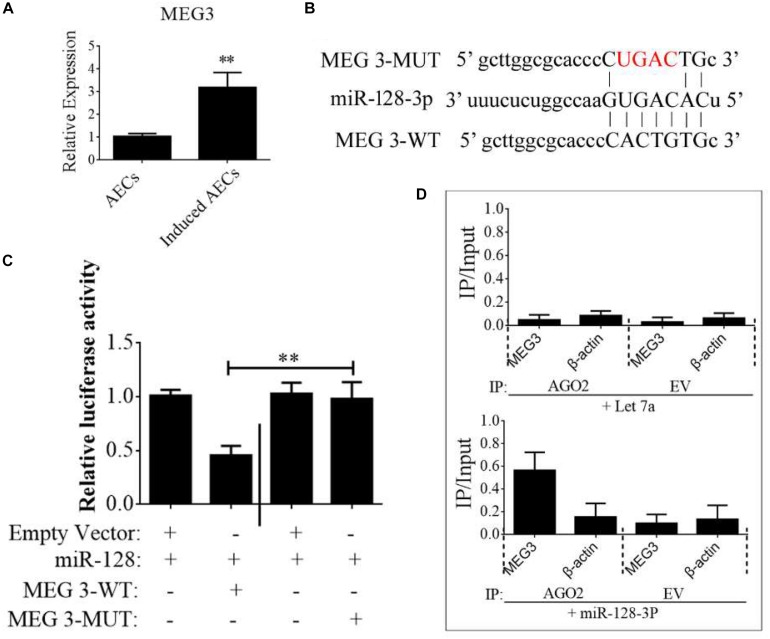
lncRNA MEG3 targets miR-128-3p in neurogenic differentiation from AECs. **(A)** The expression of MEG3 was analyzed in normal and induced AECs using RT-qPCR. **(B)** The binding site between MEG3 and miR-128-3p was predicted using bioinformatics algorithms and subsequently mutated to verify interactions. **(C)** Full-length MEG3 (WT) and sequences containing different mutated variants of the miR-128-3p binding site (MUT) were tested to determine the effect on miR-15/16 expression. The pRL-40 vector alone was used as an internal control. Results are expressed as relative luciferase activity and represent the mean ± SD of at least three replicates. **(D)** Immunoprecipitation of Myc-tagged Argonaute 2 (AGO2) from AECs co-transfected with Myc-AGO2 and either miR-128-3p or Let-7a (negative control). Empty vector (EV) served as the Myc-AGO2-related negative control. MEG3 and β-actin mRNA levels were quantified using qPCR, and relative immunoprecipitate (IP)/input (cell total RNA) values were plotted. ^∗^*P* < 0.05; ^∗∗^*P* < 0.01.

Competing endogenous RNAs are widely involved in eukaryotic development by regulating other mRNA transcripts by competing for shared miRNAs (Das et al., 2019). In this study, we assumed that the down-regulation of miR-128-3p was regulated by lncRNAs or circRNAs. Therefore, we used the StarBase bioinformatics algorithm to screen for interactions between competing endogenous (ce)RNAs and miR-128-3p to investigate the low abundance of miR-128-3p in neurogenic differentiation from AECs. The lncRNAs and circRNAs we found are listed in Supplementary Tables S2, S3. Based on this analysis, and combined with data from a previous study (Lan et al., 2018), the lncRNA MEG3 was selected as a candidate target. MEG3 is ∼1.6 kb in humans with a number of splice isoforms and evidence of retained introns creating longer transcripts. It is widely expressed early in the visceral yolk sac, embryonic ectoderm, paraxial mesoderm, epithelial ducts, skeletal muscle, cochlea, brain, and eye. We first detected MEG3 expression levels in normal and induced AECs, and showed them to dramatically increase after ATRA treatment (Figure 5A).

The binding sites between MEG3 and miR-128-3p were predicted using StarBase and are shown in Figure 5B. To investigate the interaction of miR-128-3p and MEG2 in induced AECs, miR-128-3p binding sites in MEG3 were mutated using PCR methods (Figure 5B). Then, full-length MEG3 (MEG3-WT) and MEG3 with mutated sites (MEG3-MUT) were cloned and linked downstream of a luciferase reporter gene. These constructs were co-transfected into HEK293T cells with either pre-miR-128-3p or control. Luciferase activity significantly decreased following MEG3-WT transfection relative to that in cells co-transfected with MEG3-MUT (Figure 5C). To determine whether AGO2 serves as a binding platform for MEG3 and miR-128-3p, we performed Myc-AGO2 IP in HEK293T cells containing either an AGO expression vector or an empty vector and transiently co-expressing miR-128-3p or Let-7a (negative control). The binding site of Let-7a was not found in the MEG3 sequence after analysis using bioinformatics algorithms; therefore, Let-7a was used as a negative control in this study. MEG3 levels were analyzed by qPCR of the IP products, and shown to be specifically enriched by more than 8- to 10-fold in the presence of AGO2 in miR-128-3p-transfected cells compared with the control (Let-7a-transfected cells, Figure 5D). These data suggest that miR-128-3p interacts with MEG3 in the RISC complex.

### CREB Promotes MEG3 Expression to Increase the Expression of Neuron-Specific Genes

**FIGURE 6:**
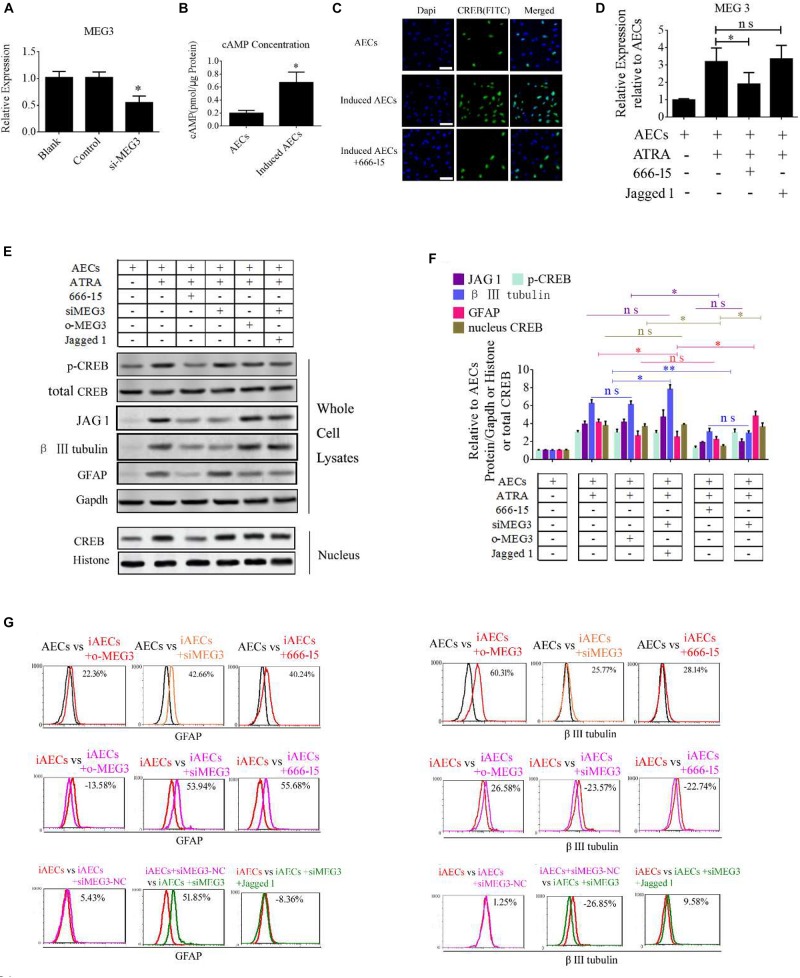
ATRA increases the expression of MEG3 through the cAMP/CREB pathway to modulate neurogenic differentiation. **(A)** A siRNA for MEG3 (si-MEG3) effectively depressed the expression of MEG3 in induced AECs (*P* < 0.05). **(B)** The concentration of cAMP was detected between normal significantly increased in induced compared with normal AECs (*P* < 0.05). **(C)** Expression of cAMP response element-binding protein (CREB) was detected under ATRA or 666-15 (a CREB inhibitor) treatment using immunofluorescence staining. **(D)** MEG3 expression was analyzed under 666-15 or Jagged 1 treatment by RT-qPCR. **(E,F)** Expression of β III-tubulin and GFAP were as assessed using Western blotting. **(G)** β III-tubulin and GFAP expression was analyzed following single or assorted overexpression of MEG3 (o-MEG3), si-MEG3 or 666-15 treatment in induced AECs. iAECs, induced AECs; siMEG3-NC, siMEG3 negative control. ^∗^*P* < 0.05; ^∗∗^*P* < 0.01; ns, non-significant.

To determine the function of MEG3 in the expression of neuron-specific genes from differentiated AECs, siRNA for MEG3 (si-MEG3) was designed and synthesized to depress the expression of MEG3 in induced AECs (Figure 6A). si-MEG3 and MEG3 were added to induced AECs, respectively, to assay the percentage of β III-tubulin and GFAP using flow cytometry. si-MEG3 dramatically increased the amount of GFAP, and inhibited the expression of β III-tubulin, while the opposite was observed after overexpressing MEG3 in induced AECs (Figure 6G). MEG3 can modulate neurogenic differentiation from AECs under ATRA treatment, but the mechanisms underlying this are unclear. Previous work (Zhao et al., 2006; Zhu et al., 2019) described how elevated cAMP levels enhanced endogenous MEG3 expression through cAMP-response element (CRE) directly binding the MEG3 promoter region. We detected the concentration of cAMP after ATRA treatment in AECs using a cAMP assay kit (competitive ELISA, Fluorometric) and combined this with our previous data (Zhao et al., 2004). We detected a marked increase in intracellular cAMP levels in induced AECs (Figure 6B). The CRE-binding protein CREB was previously shown to enter the nucleus and to combine with CRE to enhance MEG3 transcription, after elevating the concentration of cAMP (Zhao et al., 2006; Zhu et al., 2019). Here, we detected the expression of phosphorylated CREB and total CREB in whole cell lysates using Western blot and immunofluorescence. Both phosphorylated CREB and total CREB dramatically increased after ATRA treatment (Figures 6C,E). Use of the CREB inhibitor (666-15), which reduces CREB phosphorylation to inhibit its transcriptional activity independently of direct CREB or CREB-binding protein interactions (Zhang et al., 2019), significantly decreased phosphorylated CREB and total CREB in induced AECs (Figures 6E,F). 666-15 was also added to test the effect of CREB in the regulation of MEG3 transcription and was found to effectively depress MEG3 expression in induced AECs (Figure 6D). Its effects on inhibiting β III-tubulin and increasing GFAP were in line with the si-MEG3 experiment (Figures 6E–G). JAG1 was shown to block the function of si-MEG3 in neurogenic differentiation from AECs, implying that *MEG3* is an upstream regulatory gene for miR-128-3p influencing neurogenic differentiation.

**FIGURE 7:**
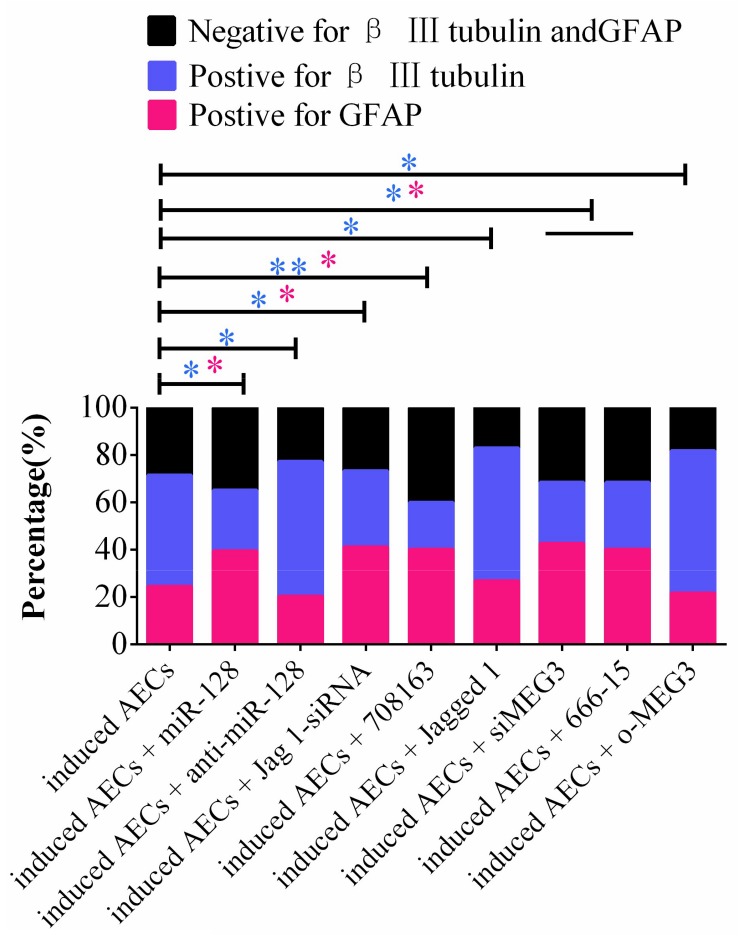
Statistical analysis of FCM data in miR-128-3p and its upstream or downstream genes affecting neurogenic differentiation. miR-128-3p reduces the amount of neurons and increases the amount of gliocytes by targeting JAG1 to prevent Notch pathway activation, which may enhance the transcription of neuron-specific markers. MEG3 increases the amount of neurons and reduces the amount of gliocytes via targeting miR-128-3p to activate the Notch pathway (^∗^*P* < 0.05; ^∗∗^*P* < 0.01; blue asterisk: neurons; pink asterisk: gliocyte).

**FIGURE 8:**
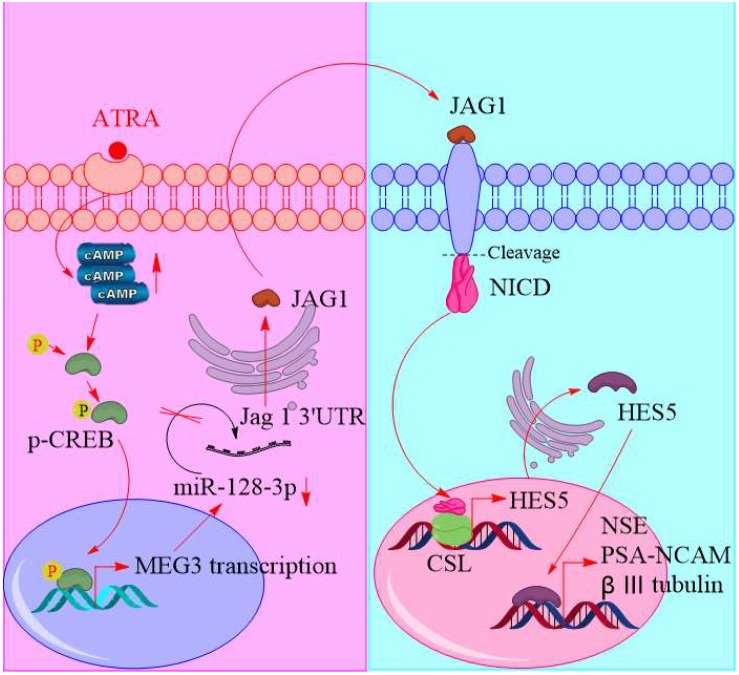
Schematic of promoting neurogenic differentiation from human amniotic epithelial cells.

Taking these findings together, the flow cytometry data show that miR-128-3p alone decreases the expression of neuron-specific genes compared with the anti-miR-128-3p group in neurogenic differentiation from AECs (*P* < 0.05). si-JAG1, JAG1, and the Notch pathway inhibitor BMS-708163 all affect neurogenic differentiation, and activation of the Notch pathway significantly increases levels of β III-tubulin (*P* < 0.05). MEG3 plays an important role in the expression of neuron-specific genes by targeting miR-128-3p to activate the Notch pathway during neurogenic differentiation. β III-tubulin levels dramatically decrease when its expression is knocked down or its transcription is inhibited (*P* < 0.05, Figures 7, 8).

## Discussion

Amniotic epithelial cells have important properties that contribute to promising potential usage in nerve regeneration. These include differentiation into all three germ layers, anti-inflammatory properties, and low immunogenicity (Bai et al., 2019). In our previous research (Gao et al., 2016), AECs demonstrated the ability to differentiate into neural cells with characteristics of functional neurons under treatment of ATRA combined with other factors. However, the underlying molecular mechanisms of ATRA were unclear, which carries potential risks for clinical applications.

The RA cell-signaling pathway plays an important role in nervous regeneration and is essential in neuronal differentiation. RA signaling has been demonstrated to promote neural differentiation in the developing embryo of zebrafish, chicken, and mice (Samarut et al., 2015; Xavier-Neto et al., 2015). In adults, this pathway’s functions are confined to a few brain regions and are involved in the maintenance of adult neurogenesis and the differentiated state of neurons. The RA pathway generates a series of physiological effects by causing a regulated cascade of gene transcription after it is activated in cells.

In our research, the Notch pathway was activated after RA treatment during neurogenic differentiation. The Notch pathway plays a critical role in many developmental processes, including proliferation, differentiation, and apoptosis. It has four classic transmembrane receptors (notch 1, notch 2, notch 3, and notch 4) that interact with specific ligands (Jagged 1 and Jagged 2) to influence cell fate. Intercellular notch receptors are cleaved by the γ-secretase complex when these ligands have interacted with a notch receptor. The NICD is cleaved several times and then trafficked into the nucleus where it interacts with special transcription factors (HES, HEY, and HERP) to increase the expression of downstream target genes. The Notch pathway is activated after RA treatment during stem cell differentiation (Osathanon et al., 2017; Kong et al., 2018), and our data reveal dramatically increased NICD and HES5 expression levels after ATRA treatment, which promote the expression of neuron-specific genes in neurogenic differentiation from AECs.

miR-128 is a brain-enriched miRNA that has been reported to participate in the regulation of neural cell differentiation and to protect neurons after FTY720 treatment. In our research in induced AECs, we found that the overexpression of miR-128 reduced neuron differentiation and increased the number of gliocytes. Gliocytes have the capacity to nourish and protect neurons *in vitro* and *in vivo*; therefore, miR-128-3p protects neurons under drug treatment by increasing the number of gliocytes. However, the molecular mechanisms involved in the low expression of miR-128-3p in our neurogenic differentiation experiments remain unclear.

Competing endogenous RNAs regulate other RNA transcripts by competing for shared miRNAs (Das et al., 2019), and lncRNAs have various biological functions including the silencing of miRNAs (Ji et al., 2018). The lncRNA MEG3 is important for growth and development and is a tumor suppressor with roles that activate p53 and prevent cell proliferation. Previous studies have found that MEG3 promoter activity is mainly attributable to a CRE site and demonstrated that proteins from the CREB family directly bind to this site. Additionally, elevated cAMP levels were reported to stimulate transcription from the MEG3 promoter and enhance MEG3 expression (Zhao et al., 2006; Zhu et al., 2019). Another study found that ATRA enhanced intracellular cAMP accumulation and CREB phosphorylation (Suwa et al., 2016). In the present study, we showed that as the concentration of intracellular cAMP accumulates, and nuclear CREB concentrations also increase after ATRA treatment in AECs. These data imply that the molecular pathway of elevated MEG3 expression involves an ATRA/cAMP/CREB/MEG3 axis. We also demonstrated the interaction between MEG3 and miR-128-3p, which is largely in agreement with other previously reported work (Lan et al., 2018). Thus, MEG3 appears to modulate the percentage of neurons versus gliocytes in neurogenic differentiation from AECs through activation of the Notch pathway to increase the expression of neuron-specific genes.

Figure 8 shows a schematic of our predicted model of this process, in which intracellular concentrations of cAMP are dramatically elevated after ATRA treatment, causing CREB phosphorylation. Phosphorylated CREB enters the nucleus to enhance lncRNA MEG3 transcription, which, as a ceRNA, interacts with miR-128-3p. JAG1 is the canonical ligand to activate the Notch pathway and is inhibited by miR-128-3p binding its 3′-UTR. JAG1 translation is therefore increased after ATRA treatment through MEG3 competitively interacting with miR-128-3p. The NICD is cleaved from the cytomembrane, translocates into the nucleus, and associates with the CSL to activate HES members, which enhances neuronal gene transcription.

## Conclusion

In this study, we elucidated the molecular pathway involved in ATRA-induced neurogenic differentiation from AECs. ATRA induction enhances the transcription of β III-tubulin, NSE, and PSA-NCAM neuron-specific genes via activation of the Notch signaling pathway. Additionally, the lncRNA MEG3, a key negative regulator of miR-128-3p, is up-regulated intracellularly by the cAMP/CREB pathway. In turn, this process directly down-regulates miR-128-3p through ceRNAs, which up-regulates JAG1 expression, the target of miR-128-3p, and activates the Notch signaling pathway. Our study, which particularly focuses on the involvement of miR-128-3p in neuron differentiation from AECs, may assist in the future development of effective cell transplant therapies for the treatment of neurodegenerative disease.

## Data Availability Statement

The raw data supporting the conclusions of this article will be made available by the authors, without undue reservation, to any qualified researcher.

## Ethics Statement

The animal study was reviewed and approved by the Ethics Committee of Jining Medical University (2017-JZ-003).

## Author Contributions

YG performed the cell differentiation, Western blotting, and FCM, and drafted the manuscript. SD, XZ, and RZ cultured the cells. CB performed the RNAi, cultured the cells, and reviewed the manuscript. XL analyzed the data and reviewed the manuscript. WY and GW participated in its design and coordination.

## Conflict of Interest

The authors declare that the research was conducted in the absence of any commercial or financial relationships that could be construed as a potential conflict of interest.

## Footnotes

1 http://jaspar.genereg.net/

2 http://www.targetscan.org/vert_72/
