# Supplementary figures and images for: Long Non-coding RNA Maternally Expressed 3 Increases the Expression of Neuron-Specific Genes by Targeting miR-128-3p in All-Trans Retinoic Acid-Induced Neurogenic Differentiation From Amniotic Epithelial Cells
Source: Front Cell Dev Biol. 2019 Dec 23;7:342. doi: 10.3389/fcell.2019.00342 (PMC6936004; doi:10.3389/fcell.2019.00342)

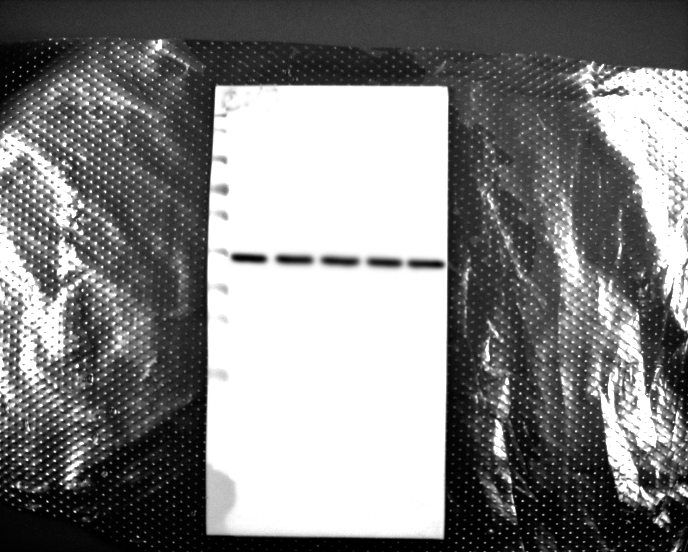

Supplement: Supplementary file 1 [file Presentation_1.ZIP › Figure 2A-GAPDH.bmp]

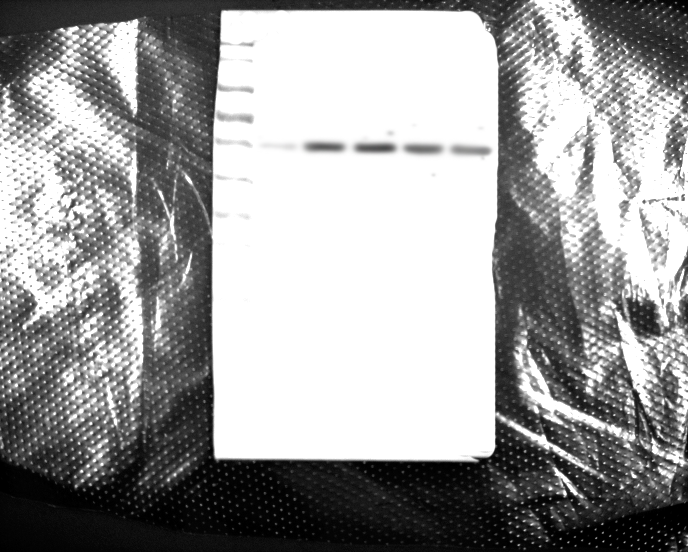

Supplement: Supplementary file 1 [file Presentation_1.ZIP › Figure 2A-GFAP.bmp]

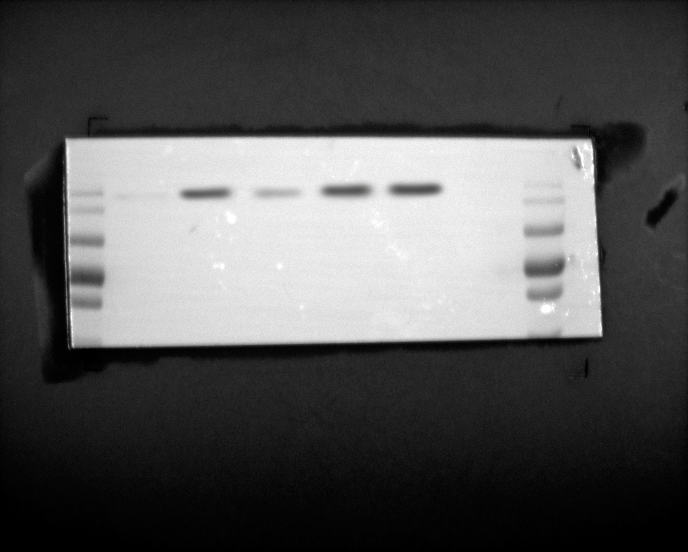

Supplement: Supplementary file 1 [file Presentation_1.ZIP › Figure 2A-Jag 1.bmp]

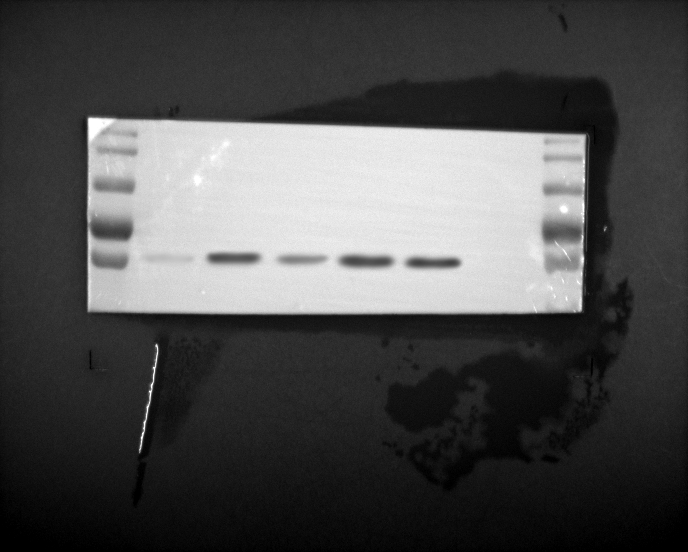

Supplement: Supplementary file 1 [file Presentation_1.ZIP › Figure 2A-tubulin.bmp]

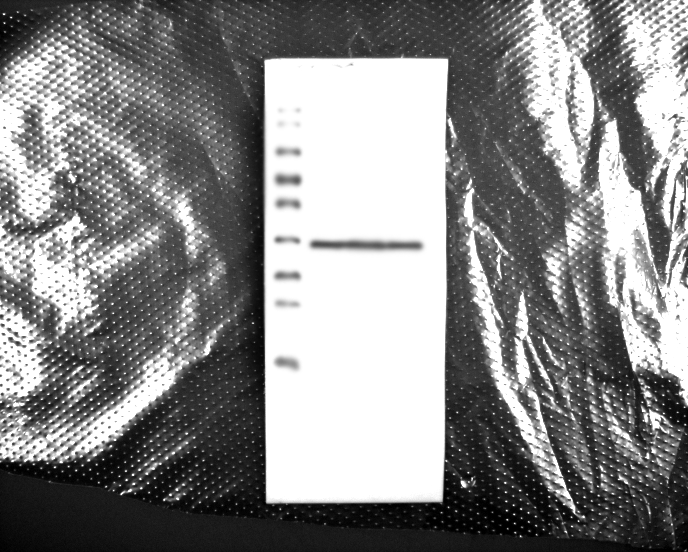

Supplement: Supplementary file 1 [file Presentation_1.ZIP › Figure 2E-Gapdh.bmp]

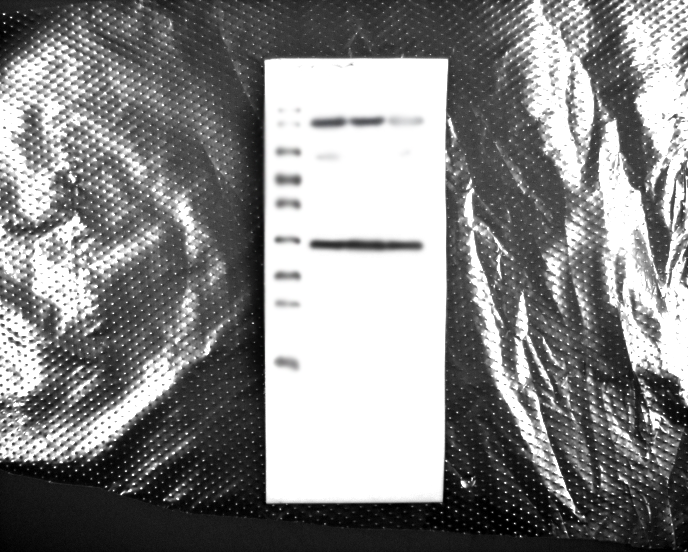

Supplement: Supplementary file 1 [file Presentation_1.ZIP › Figure 2E-JAG 1.bmp]

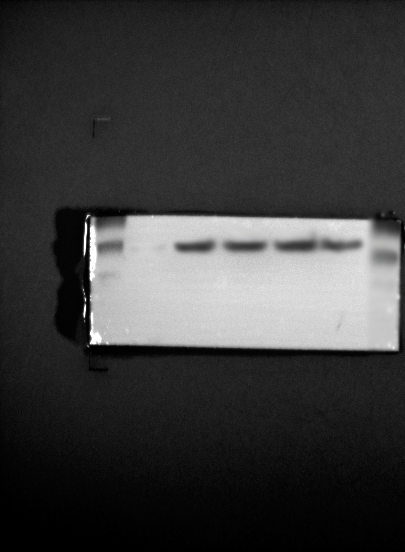

Supplement: Supplementary file 1 [file Presentation_1.ZIP › Figure 3C-GFAP.bmp]

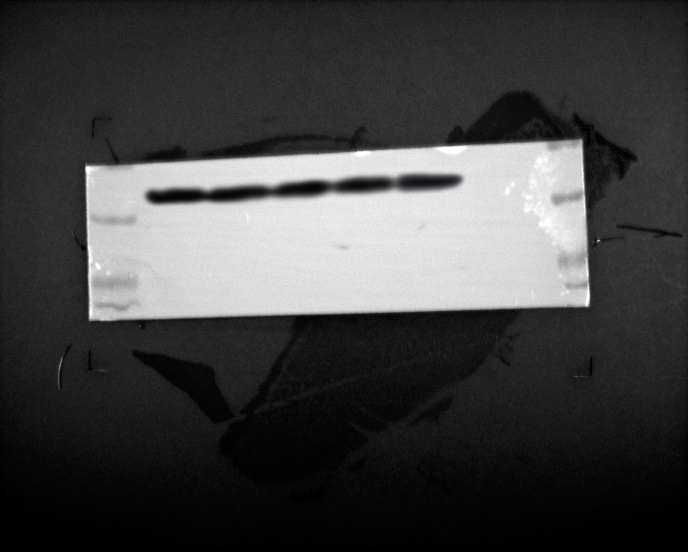

Supplement: Supplementary file 1 [file Presentation_1.ZIP › Figure 3C-Gapdh.bmp]

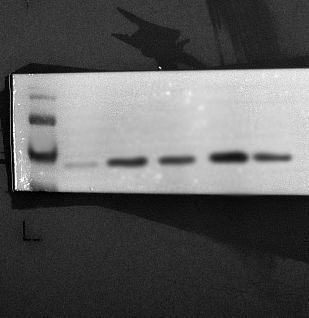

Supplement: Supplementary file 1 [file Presentation_1.ZIP › Figure 3C-HES5.bmp]

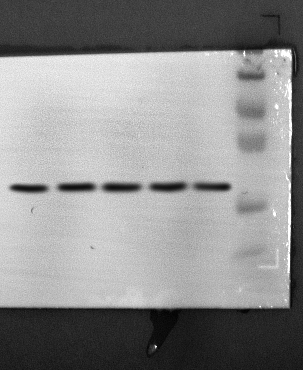

Supplement: Supplementary file 1 [file Presentation_1.ZIP › Figure 3C-Histone.bmp]

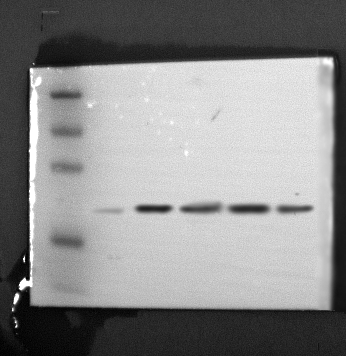

Supplement: Supplementary file 1 [file Presentation_1.ZIP › Figure 3C-NICD.bmp]

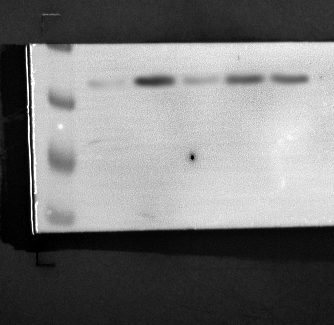

Supplement: Supplementary file 1 [file Presentation_1.ZIP › Figure 3C-a┬ó≤ tubulin.bmp]

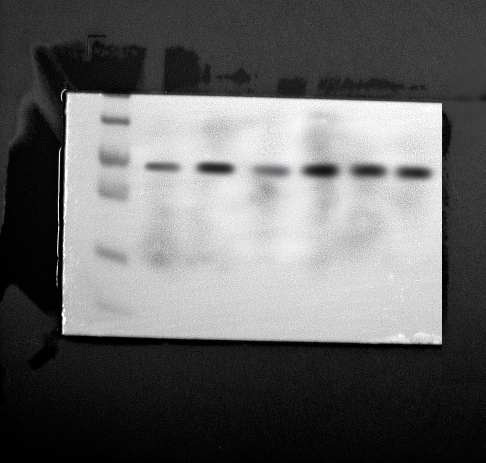

Supplement: Supplementary file 1 [file Presentation_1.ZIP › Figure 6E-CREB.bmp]

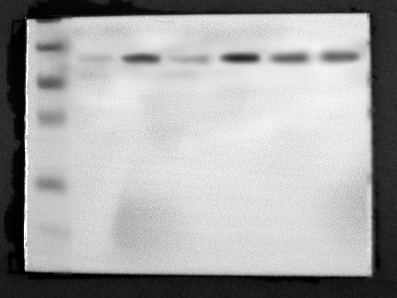

Supplement: Supplementary file 1 [file Presentation_1.ZIP › Figure 6E-GFAP.bmp]

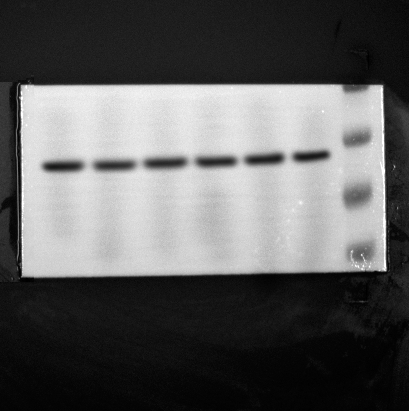

Supplement: Supplementary file 1 [file Presentation_1.ZIP › Figure 6E-Gapgh.bmp]

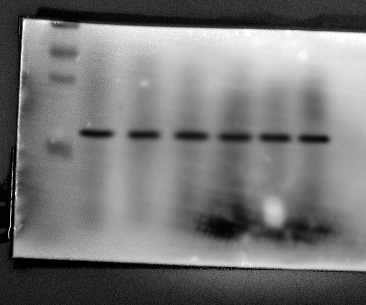

Supplement: Supplementary file 1 [file Presentation_1.ZIP › Figure 6E-Histone.bmp]

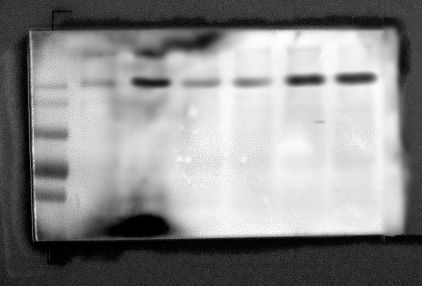

Supplement: Supplementary file 1 [file Presentation_1.ZIP › Figure 6E-JAG1.bmp]

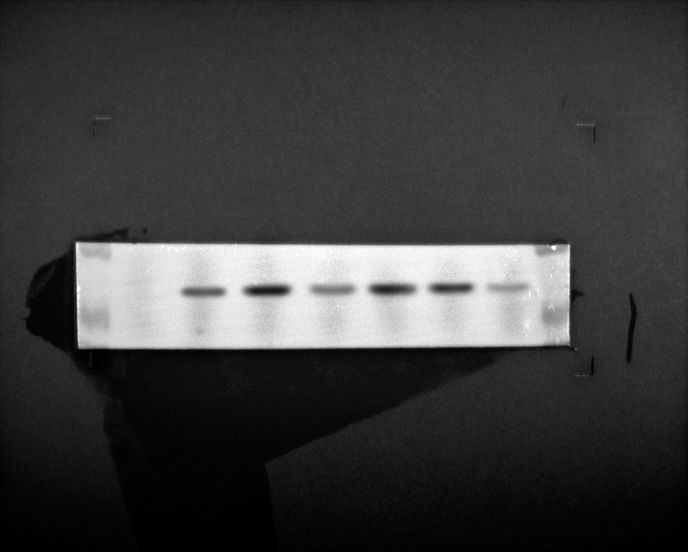

Supplement: Supplementary file 1 [file Presentation_1.ZIP › Figure 6E-p-CREB.bmp]

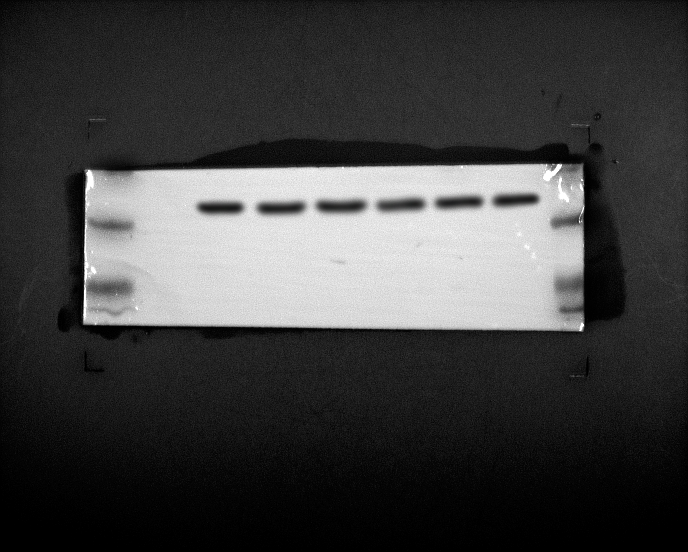

Supplement: Supplementary file 1 [file Presentation_1.ZIP › Figure 6E-total CREB.bmp]

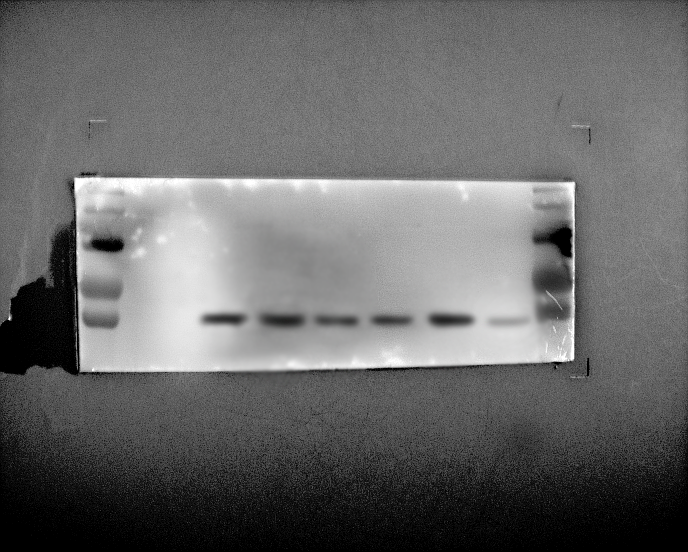

Supplement: Supplementary file 1 [file Presentation_1.ZIP › Figure 6E-a┬ó≤ tubulin.bmp]
